# Exploration of neuroprotective effect from *Coriandrum sativum* L. ethanolic seeds extracts on brain of obese rats

**DOI:** 10.1038/s41598-024-51221-5

**Published:** 2024-01-05

**Authors:** Novi Silvia Hardiany, Putri Krishna Kumara Dewi, Syarifah Dewi, Bimo A. Tejo

**Affiliations:** 1 Department of Biochemistry and Molecular Biology, Faculty of Medicine, Universitas Indonesia, Jakarta, 10430 Indonesia; 2 Center of Hypoxia and Oxidative Stress Studies, Faculty of Medicine, Universitas Indonesia, Jakarta, 10430 Indonesia; 3 Master Program in Biomedical Sciences, Faculty of Medicine, Universitas Indonesia, Jakarta, 10430 Indonesia; 4 Medical Biochemistry Division, Department of Biomedical Science, Faculty of Medicine, Universitas Pendidikan Ganesha, Bali, 81116 Indonesia; 5 Department of Chemistry, Faculty of Science, Universiti Putra Malaysia, 43400 Serdang, Malaysia

**Keywords:** Biochemistry, Molecular biology, Neuroscience

## Abstract

In this study, the potential neuroprotective ability of coriander seeds (*Coriandrum sativum* L.) ethanolic extract (CSES) as a neuroprotectant agent in the brains of high-fat diet-induced obese rats was analyzed. The study investigated how CSES impacts oxidative stress markers (i.e., malondialdehyde/MDA, glutathione/GSH and catalase), inflammation marker (i.e., Interleukin-6/IL-6), cellular senescence markers (i.e., senescence-associated β-galactoside/SA-β-Gal activity and p16), brain damage marker (i.e., Neuron-specific Enolase/NSE), and neurogenesis markers (i.e., mature Brain-derived Neurotropic Factor/BDNF, pro-BDNF, and mature/pro-BDNF ratio). Male adult Wistar rats were fed a high-fat diet and given CSES once daily, at 100 mg/kg body weight, for 12 weeks. CSES significantly reduced MDA concentration (p = < 0.001), SA-β-Gal activity (p = 0.010), and increased GSH concentration (p = 0.047) in the brain of obese rats; however, the decrease of IL-6, NSE, and p16 as well as the increase of catalase specific activity and BDNF expression were not significant. Moreover, the mature/pro-BDNF ratio was significantly higher in the brains of non-obese rats, both given the control diet and the high-fat diet compared to the control. Our results suggest that obese rats benefited from consuming CSES, showing improved oxidative stress levels, reduced cellular senescence and increased endogenous antioxidants, making CSES a potential neuroprotective agent.

## Introduction

Obesity and overweight have become global problems in the last 10 years^1^. Increased intake of high sugar, salt, and fat has a vital role in the incidence of obesity in the population^2^. Obesity leads to chronic systemic inflammation and increased circulating free fatty acid/FFA that affects all organ systems, including the nervous system. Obesity causes inflammation in the brain, impairs mitochondrial function, and increases the production of reactive oxygen species (ROS) and oxidative stress. Continuous oxidative stress will trigger the senescence state. Senescence is a response to cell stress characterized by permanent cell growth arrest and changes in gene expression and cell morphology^3^. These impairments can lead to various issues, including impaired neuronal plasticity, decreased neurogenesis ability, neuronal death, and impaired neuronal function manifested as cognitive, behavioural, and motor-sensory problems^4,5^. In cellular stress due to inflammation, trauma, or disruption of cell homeostasis, brain-derived neurotropic factor/BDNF is secreted to maintain neuron survival and neurogenesis^6^. BDNF reacted with ROS as a protective agent against oxidative stress-induced damage to the central nervous system^7,8^.

Various therapeutic modalities have been investigated to address obesity and its problems. Natural antioxidants and anti-inflammatory agents like polyphenols and flavonoids are being studied as potential therapies for obesity and long-term high-fat diets^9^. Coriander (*Coriandrum sativum *L.) is a promising natural therapeutic candidate due to its reported antimicrobial, antioxidant, anti-inflammatory, antilipidemic, antidiabetic, anxiolytic, and sedative-hypnotic activities, as well as its ability to cross the blood-brain barrier^10,11^. Coriander leaves have been shown to improve memory deficit in mice models^12^. The extract of coriander seeds slowed down aging memory decline in SAMP8 mice by increasing neurofilament-light mRNA and decreasing neuronal nitric oxide synthase/NOS mRNA in their frontal lobe^11^. Our previous study proved that coriander seed extract can reduce triglyceride, eliminate oxidative stress, and inhibit cellular senescence in the liver of obese rats^13^.

Obesity is known to trigger chronic inflammation, prolonged oxidative stress, cellular senescence, and degenerative brain diseases. Consequently, it is imperative to delve into the potential of natural phytochemical agents, such as coriander, which exhibit antioxidant, anti-inflammatory, and antilipidemic properties, for further study as therapeutic modalities to combat obesity and its diverse adverse effects. Therefore, this study aimed to investigate the effects of coriander seed ethanol extract on brain inflammation, oxidative stress, cellular senescence, and brain damage in rats with obesity induced by a high-fat diet. This study also examined the extract's impact on serum neuron-specific enolase/NSE levels as a brain damage marker and brain neurogenesis via BDNF expression.

## Results

### Effect of CSES on IL-6, malondialdehyde/MDA, glutathione/GSH, and catalase-specific activity

**Figure 1:**
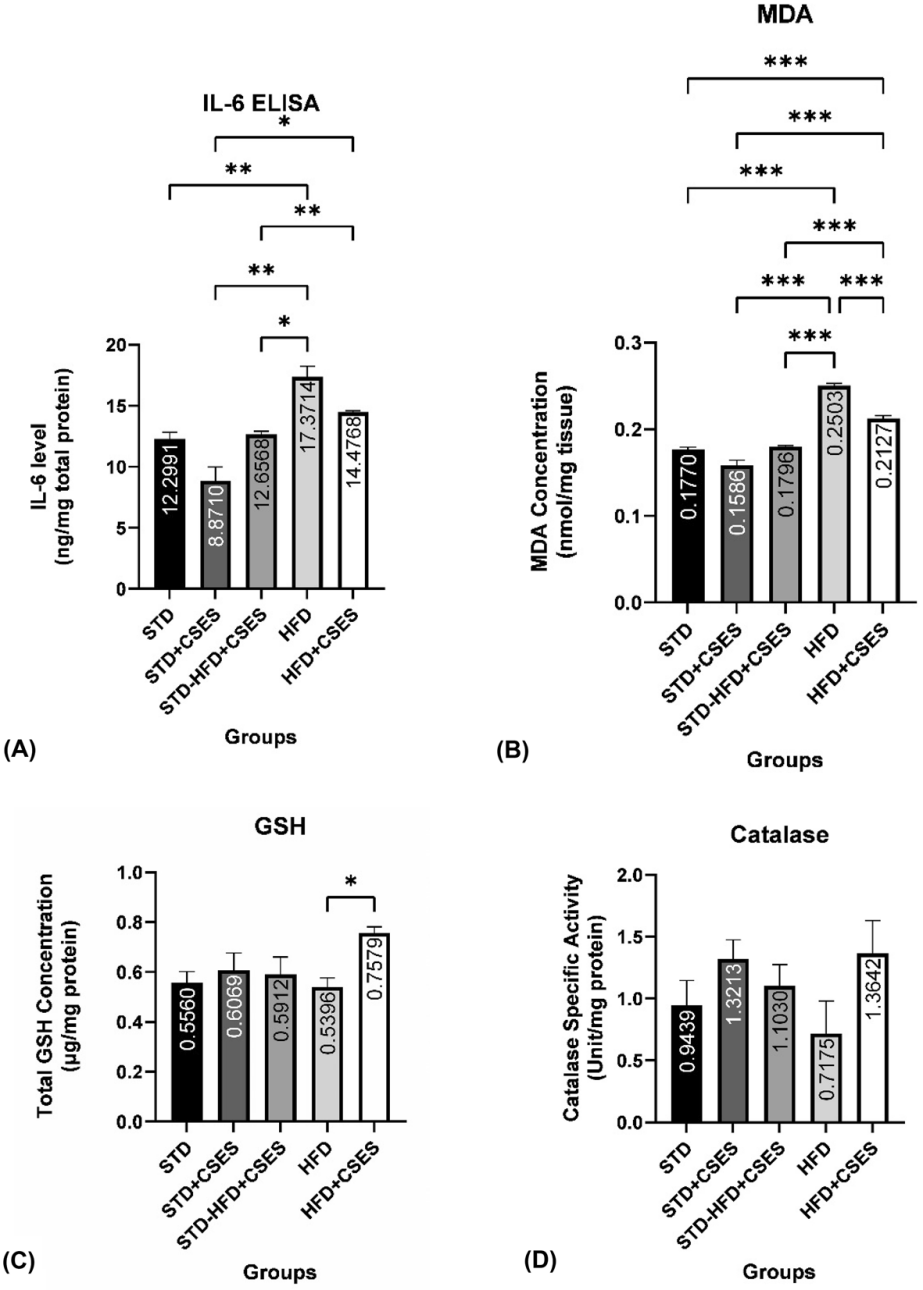
The effect of CSES on pro-inflammatory cytokine concentration and oxidative stress state in the rat brain. (**A**) Comparison of IL-6 level between groups. The impact of daily CSES  on lowering brain IL6. (**B**) MDA concentration between groups and the effect of daily CSES on MDA concentration. (**C**) The concentration of GSH between groups and the impact of daily CSES on GSH concentration. (**D**) Comparison of catalase-specific activity between groups. *STD:* standard control, *STD + CSES:* standard + coriander seeds extract, *STD-HFD + CSES:* standard diet for 12 weeks, then replaced with a high-fat diet + coriander seeds extract for another 12 weeks, *HFD:* obese control, *HFD + CSES*: obese + coriander seeds extract. IL6 and MDA: Brown-Forsythe and Welch One-way ANOVA post hoc Dunnett T3. GSH and catalase-specific activity: One-way ANOVA post hoc Tukey. Graph mean ± SEM *p < 0.05; **p < 0.01; ***p < 0.001.

To evaluate the initial stages of the damage caused by obesity, we studied the pro-inflammatory cytokine IL-6, MDA, and endogenous antioxidants GSH and catalase-specific activity in the brain. The significant increase in IL-6 (p = 0.008, Fig. 1A) and MDA levels (p = < 0.001, Fig. 1B), along with the decreasing tendency of the endogenous antioxidant GSH (p = 1.0, Fig. 1C) and catalase (p = 0.900, Fig. 1D) in the obese control group compared to the control group, proved that obesity-induced high-fat diet leads to an increased stress state due to persistent uneliminated oxidants in the brain. These results indicate that obesity and high-fat intake cause inflammation and oxidative stress in the rat brain.

In this study, the CSES groups exhibited a significant decrease in MDA levels (p = < 0.001, Fig. 1B) and a significant increase in total GSH levels (p = 0.047, Fig. 1C) compared to the control groups**.** These results align with decreased IL-6 (p = 0.08, Fig. 1A) and a trend of increased catalase-specific activity (p = 0.600, Fig. 1D) in CSES groups compared to controls. All results indicated a net reduction in brain oxidative stress and inflammation in CSES groups.

### Effect of CSES on cellular senescence markers

**Figure 2:**
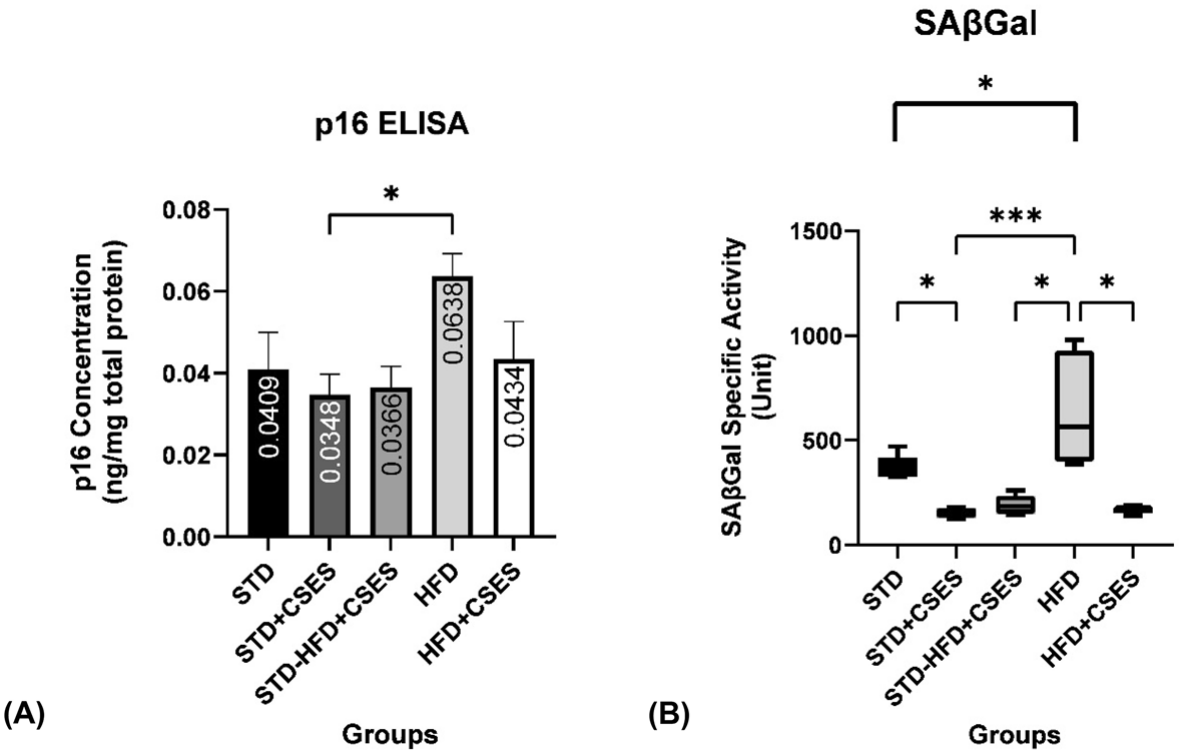
The effect of CSES on the development of premature cellular senescence in the brain of obese rats (**A**) Comparison of SA-β-Gal activity between groups and the daily CSES effect on SA-β-Gal activity. (**B**) p16^INK4a^ protein concentration between group's and daily CSES effect on each group p16^INK4a^ protein level. *STD:* standard control, *STD + CSES*: standard + coriander seeds extract, *STD-HFD + CSES*: standard diet for 12 weeks, then replaced with a high-fat diet + coriander seeds extract for another 12 weeks, *HFD:* obese control, *HFD + CSES*: obese + coriander seeds extract. SA-β-Gal activity: Kruskal-Wallis non-parametric analysis + Bonferroni correction. p16^INK4a^: One-way ANOVA post hoc Tukey. Graph mean ± SEM *p < 0.05; ***p < 0.001.

Our results showed a significant increase in SA-β-Gal activity in the obese control group compared to the control group (p = 0.001). This result aligns with the trend of increased expression of p16^INK4a^ protein in the obese control (p = 0.188) compared to the standard control (Fig. 2A). They indicate senescence-like behavior and the possibility of premature senescence in the brains of obese rats.

All groups given CSES showed a significant decrease in SA-β-Gal activity (Fig. 2B) compared to the control groups (p = 0.010). These results align with the decreased trend of p16^INK4a^ protein levels in all coriander groups compared to controls (p = 0.242).

### Effect of CSES on neuron-specific enolase (NSE) concentration and BDNF expression

**Figure 3:**
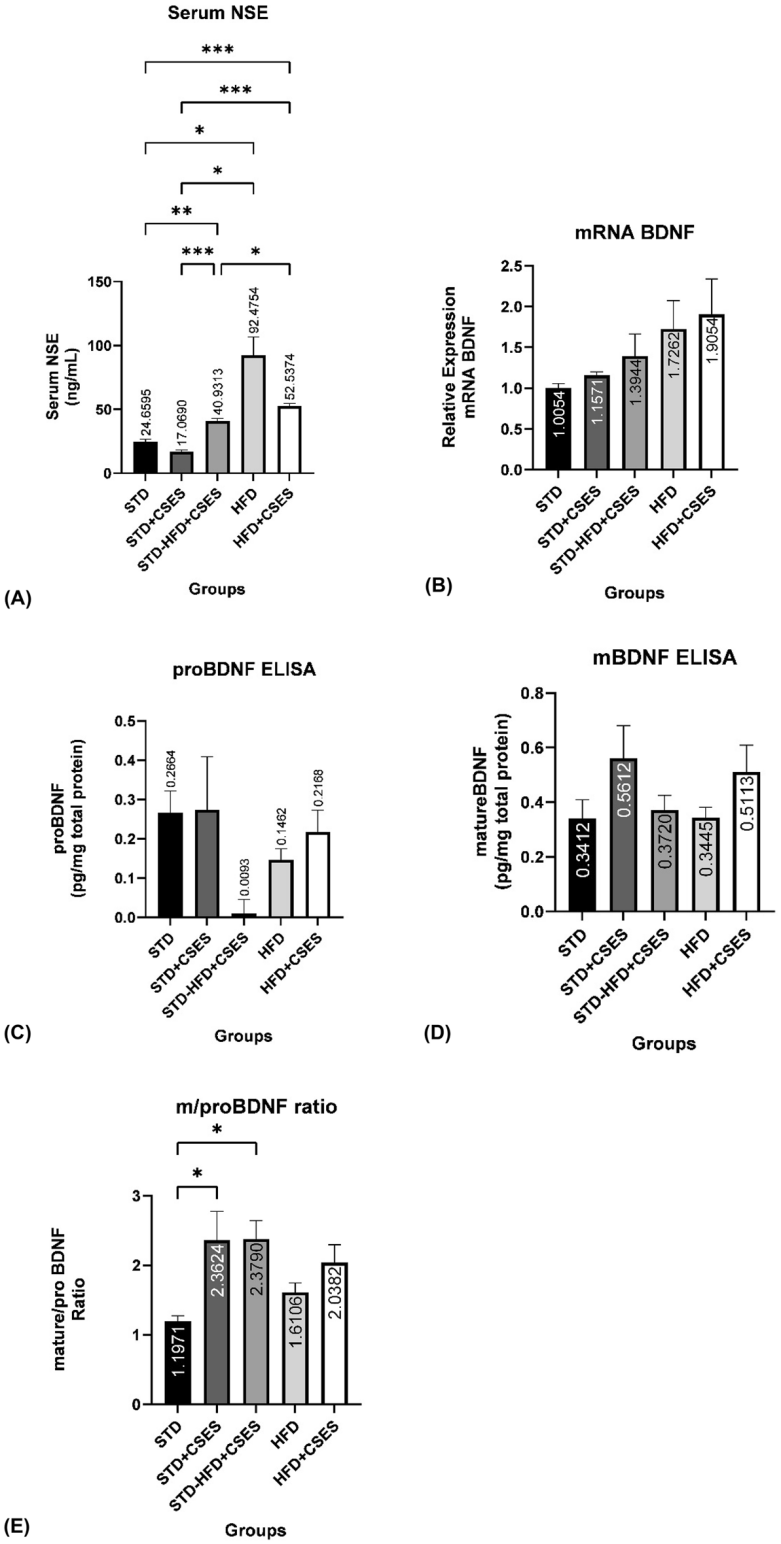
The effect of CSES on the brain damage process and neurogenesis ability in the brain of obese rats. (**A**) Comparison of serum NSE concentration between groups and CSES effect on serum NSE level in obese rats. (**B**) BDNF mRNA relative expression in each group and the impact of CSES on the expression of BDNF mRNA. (**C**) The concentration of pro-BDNF in obese rats' brain tissue and the effect of CSES on pro-BDNF protein concentration. (**D**) The concentration of mature-BDNF protein in obese brain tissue and the impact of CSES on mature-BDNF concentration. (**E**) The ratio of mature-BDNF to pro-BDNF concentration between groups indicates the effect of CSES on the maturation of BDNF protein in obese brain tissue. *STD*: standard control, *STD + CSES:* standard + coriander seeds extract, *STD-HFD + CSES:* standard diet for 12 weeks, then replaced with a high-fat diet + coriander seeds extract for another 12 weeks, *HFD*: obese control, *HFD + CSES*: obese + coriander seeds extract. Serum NSE, mRNA BDNF expression, and mBDNF protein concentration: Brown-Forsythe and Welch One-way ANOVA post hoc Dunnett T3. Pro-BDNF and ratio mature to pro-BDNF: One-way ANOVA post hoc Tukey. Graph mean ± SEM *p < 0.05; **p < 0.01; ***p < 0.001.

There was a significant serum NSE increase in the high-fat diet control group compared to the control group (p = 0.032), indicating an increased brain damage process in the obese control compared to the control. Meanwhile, all CSES-treated groups showed a decline in serum NSE levels (p < 0.001, Fig. 3A). For BDNF expression, there were no significant differences observed, both in mRNA (Fig. 3B) and protein levels, including mature-BDNF (Fig. 3C) and pro-BDNF (Fig. 3D). However, the mature/pro-BDNF ratio experienced a significant increase in non-obese rats, whether they were on a control diet (p = 0.044) or a high-fat diet (p = 0.040), compared to the control (Fig. 3E).

## Discussion

High-fat diet that leads to obesity can result in heightened cellular stress in the brain due to excess oxidants that cannot be purged, eventually impairing neuronal function; therefore, exploration of promising natural neuroprotective candidate such as coriander is important. Our results proved that elevated IL-6 and MDA levels and decreased glutathione and catalase-specific activity were found in the obese group compared to the control group. Our results affirm various studies showing increased oxidative stress in obesity and high-fat diets. Zhang et al. found that feeding a high-fat diet to Sprague-Dawley rats for five months activated the nuclear factor-κB pathway and increased the production of ROS and NOS in the cerebral cortex, contributing to neuronal damage and cognitive decline^14^. Previous work by Treviño et al. on a group of Wistar rats fed a high-calorie diet for 13 weeks found increased ROS accompanied by memory impairment^15^.

A comparison of the obese and coriander groups in this study showed a significant decrease in MDA levels in the coriander group, accompanied by a significant increase in GSH levels. They caused a net decrease in brain oxidative stress in the coriander group compared to the obese group. Cioanca et al. found that inhalation of essential oil from coriander seed (1% and 3%) can improve the state of oxidative stress in mice. This inhalation was shown to increase glutathione peroxidase levels and decrease MDA levels^16^. The production of GSH and other antioxidants in the brain is strongly influenced by the transcription factor nuclear factor erythroid 2-related factor 2/Nrf2. Nrf2 is a transcription factor produced in response to the presence of ROS, so its activity will increase as ROS increases. The uniqueness of the relationship between Nrf2 and ROS is that small and medium amounts of ROS will activate Nrf2, while high ROS conditions will inhibit Nrf2 activation, leading cells to apoptosis. Recent studies have also found that plant phenols act as antioxidants by enhancing Nrf2 activation^17^. This process explains the most significant increase in GSH in the group with the highest ROS-inducing factor (obesity), who also received a phenolic compound (coriander seed extract)^18^.

Chronic inflammation and oxidative stress in obesity can lead to premature organ senescence. Our result indicated senescence-like behavior and the possibility of premature senescence in the brains of obese rats, which was proven by increasing Senescence-associated β-Galactosidase/SA-β-Gal and p16^INK4a^. SA-β-Gal is a lysosomal hydrolase enzyme specifically active at pH 6 in senescent cells and has long been used as a senescence marker^18^. The p16^INK4a^ protein is called a "guardian" protein due to its function in senescence formation and ensuring that the cell remains in a state of senescence. The presence of p16^INK4a^ in cells indicates that the cells are already in a state of maintenance senescence^19^. These senescence progenitors can initiate immune cell infiltration, increase the body's pro-inflammatory status, and lead to an ongoing inflammation-oxidative stress-senescence cycle^20^.

The administration of coriander could reduce brain cellular senescence, which was proven by a significant decrease in SA-β-Gal activity in all groups treated with the extract compared to the control group. There was a tendency to decrease p16^INK4a^ levels in the coriander groups compared to the control. At a dose of 100 mg/kg BW/day used in this study, the decrease in p16^INK4a^ protein levels found was insignificant. There has been no study that examines the effect of coriander extract on senescence markers, as well as on premature senescence induced by a high-fat diet. Mima et al. examined the effect of coriander seed extract on memory improvement in SAMP8 aging model mice. There was an increase in nitric oxide (NO) production, which can produce ROS and NOS in SAMP8 mice without extract supplementation. Administration of coriander seed extract at a dose of 200 mg/kg BW/day for 12 weeks improved spatial memory and decreased nNOS mRNA levels in the frontal lobe of rats and not in the hippocampus^11,21^, indicating different functions and responses in various rat brain parts to positive and negative stimuli. The significant decrease in SA-β-Gal activity and the tendency to decrease p16^INK4a^ protein expression in this study indicate the improvement of senescence cell clearance function by immune cells, improving organ metabolic balance, and contributing to reducing local and systemic pro-inflammatory states in experimental animals.

The increase of brain cellular senescence in the obese group was accompanied by increased brain damage, that was proven by increasing NSE. Increased NSE levels are associated with oxidative damage as a diagnostic marker in numerous neurodegenerative diseases such as Huntington's disease, Parkinson's disease, Alzheimer's disease, and amyotrophic lateral sclerosis^22–25^. Enolase levels increase in astrocytes and microglia after cerebrovascular incidents, suggesting NSE's role in neuroinflammatory pathologies^26^. The administration of coriander tended to reduce NSE in obese rats; however, it was not significant. Moreover, coriander treatment in this study has not affected neurogenesis due to no significant change in BDNF expression. BDNF plays an essential role in developing neuronal circuits, formation and maintenance of neuronal morphology, brain and synapse architecture, and plasticity of neural networks^27^. In performing its function, BDNF binds to two classes of cell surface receptors, the p75 neurotrophin receptor (p75NTR) and the tyrosine kinase receptor B (TrkB)^21^. Pro-BDNF mainly binds to p75NTR, part of the tumor necrosis factor superfamily, and induces neuronal apoptosis^28^. Mature-BDNF mainly binds to TrkB receptors, triggering neuronal development and differentiation, cell survival, long-term potentiation, and synapse plasticity. Reduced circulating mature-BDNF levels and a diminished mature-BDNF/pro-BDNF ratio in individuals with depression and bipolar disorder prove to be more responsive biomarkers than a reduction in total BDNF levels. Furthermore, a decreased mature-BDNF/pro-BDNF ratio is a more discerning indicator than total BDNF levels in patients displaying early symptoms of Parkinson's disease^29^.

Statistical calculations found a significant increase in the ratio of mature to pro-BDNF in the coriander preventive group and the standard feed plus extract group compared to the standard feed without extract, showing that coriander stimulated neurogenesis in non-obese conditions. The maturation of pro-BDNF into mature-BDNF in adult organisms is an essential process in the function of neuronal plasticity^30^. Several studies indicate impaired maturation and a decreased mature-BDNF/pro-BDNF ratio as early indicators of neurodegenerative disease development^31^. Based on the research referring to the importance of pro-BDNF maturation into mature-BDNF above, the increase in the ratio of the amount of mature-BDNF compared to pro-BDNF in the group given coriander extract compared to the control group indicates a positive effect of coriander extract on the regulation of BDNF protein expression in a high-fat diet.

**Figure 4:**
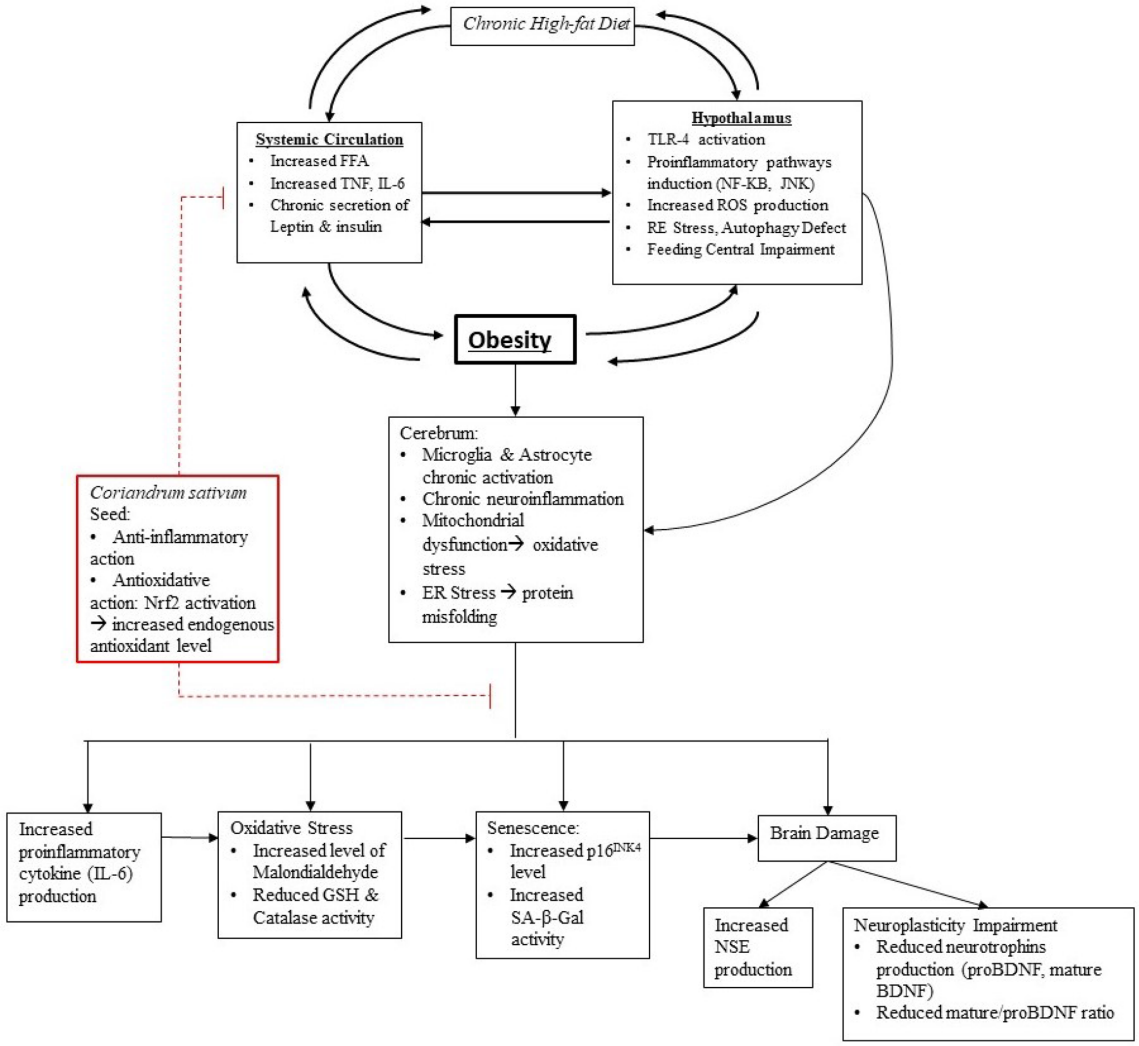
Relationship between the brain, obesity, and chronic persistent neuronal injury that leads to the development of neurodegenerative disease (black arrow lines). The hypothalamus was affected by a high-fat diet long before obesity developed. Persistent hypothalamic neuroinflammation due to a chronic high-fat diet and its resultant disruption of feeding centers play an important role in the initiation of obesity. Neuronal injury in the cerebrum leads to increased proinflammatory cytokine IL-6, increased oxidative stress markers, increased markers of premature senescence, reduced production and maturity of BDNF protein and increased marker of neuronal injury (NSE). *Coriandrum sativum* seed extract and its anti-inflammatory and antioxidant actions work to reduce inflammation, reduce oxidant levels, and increase endogenous antioxidant levels both systemically and in brain tissue (red dash lines), thus inhibiting prolonged neuronal injury and its advanced adverse effects. *FFA:* free fatty acid, *TNF:* tumor necrosis factor, *TLR:* toll-liked receptors, *TG*: triglycerides, *ER:* endoplasmic reticulum, *SASP:* senescence-associated secretory phenotype.

The study posits that the neuroprotective mechanism of *Coriandrum sativum* L. extract involves the inhibition of oxidative stress and inflammation processes, both systemically and in brain tissue. The polyphenolic compounds in coriander, particularly flavonoids, serve as antioxidants, mitigating the accumulation of reactive oxygen species (ROS) in obese conditions. Furthermore, the extract enhances endogenous antioxidants, such as glutathione, potentially through the activation of Nrf2. This dual action—reducing oxidative stress and boosting endogenous antioxidants—prevents cellular senescence, ultimately leading to improved brain health and neurogenesis. Figure 4 summarizes the role of Coriandrum sativum extract against the neural adverse effects of obesity.

In conclusion, the 12-week administration of 100 mg/kg body weight of coriander successfully mitigated brain stress linked to obesity by diminishing oxidative stress, inflammation, and cellular senescence, yet did not trigger neurogenesis. The limitation of this study was the active compound in coriander seeds, responsible for reducing oxidative stress and inflammation and mitigating cellular senescence in the brain, remains unidentified. Therefore, isolating the active compound is crucial for a more meaningful and in-depth investigation. Additionally, it is essential to note that the coriander seed extract was not administered in multiple doses. Subsequent research endeavors could delve into optimizing the biological effects, primarily on neurogenesis, through administering higher doses exceeding 100 mg/kg body weight. Moreover, to substantiate advancements in brain damage recovery, future research should incorporate histopathological analysis alongside the current biochemical parameter measurements, ensuring a comprehensive evaluation of improvement.

## Methods

### Ethics

This study complied with the Ethics Committee of the Faculty of Medicine Universitas Indonesia's animal research ethics requirements. A statement letter passing the ethical review of this study was issued on 27th February 2023 by the Health Research Ethics Committee, Faculty of Medicine, Universitas Indonesia, with number KET-249/UN2.F1/ETIK/PPM.00.02/2023. All procedures adhered to the appropriate guidelines and regulations and the methods were reported in compliance with the ARRIVE guidelines for documenting animal experiments. Experimental research and field studies on plants (either cultivated or wild), including the collection of plant material, were performed in accordance with relevant institutional, national, and international guidelines and legislation.

### Design

This was an experimental study using 29 adult male Wistar rats (*Rattus norvegicus*) as subjects. This study aimed to investigate the ability of *Coriandrum sativum* L. extract to counteract the adverse effects of obesity on the brains of obese rats. This study quantitatively measured several parameters in the subjects' brain tissue and blood serum. Parameters measured were the prooxidant level (malondialdehyde) and endogenous antioxidant level (reduced glutathione and catalase-specific activity); interleukin 6 (IL-6) proinflammatory cytokine level; cellular senescence markers (SA-β-Gal activity and p16^INK4a^ protein levels); brain cellular damage marker (serum neuron-specific enolase levels) and neurogenesis marker (proBDNF and matureBDNF protein level, mature/proBDNF ratio).

### Coriander seeds (*Coriandrum sativum* L.) ethanolic extraction (CSES)

Coriander dried fruit (seeds) were obtained from the Research Centre for Medicinal Plants and Spices, Ministry of Agriculture of Indonesia. The Herbarium Depokensis conducted authentication of coriander seed extract at the Department of Biology, Faculty of Mathematics and Natural Sciences, Universitas Indonesia. The identification confirmed that the coriander seed extract belonged to the species *Coriandrum sativum* L. and the family Apiaceae. To prepare the extract, coriander seeds were mashed and then subjected to maceration using ethanol solvent, allowing them to stand for 24 hours. The resulting solution was filtered and evaporated to obtain the ethanol extract. This extract underwent a freeze-drying process, giving it a denser form that was easily soluble in water and more durable. The extraction process was repeated six times, and the final extract was stored in a refrigerator at 4 °C until needed^13^.

CSES was administered to experimental animals daily from week 13 to week 24 (12 weeks duration). The extract was administered orally (sonde) for groups 2, 3, and 4 at 100 mg/kg body weight/day in 1 cc/0.5 kg body weight volume^13,32^.

### Animal procedures

Subjects were adult male Wistar rats (Rattus novergicus), 8-10 weeks of age, with an initial weight of around 200 g. Experiments were conducted over 24 weeks, and CSES was given in the last 12 weeks. Subjects were euthanized at week 25, and the brain tissue of each subject was obtained for further laboratory investigation.

Subjects were divided into five groups, each comprising six rats, except for the control group (STD), which consisted of five rats. Various treatments were administered to the groups as outlined below:

1. STD (n = 5): the group was given only standard chows (standard control)
2. STD + CSES (n = 6): group given standard chow + CSES
3. STD-HFD + CSES (n = 6): the group was given standard chow for the first 12 weeks, then switched to a high-fat diet/HFD + CSES for the last 12 weeks
4. HFD (n = 6): the group was given only HFD (obese control)
5. HFD + CSES (n = 6): group given HFD + CSES.

The nutritional status of the subjects was determined at week 12 of treatment through the Lee Index and blood lipid profile^13^. Necropsy was performed at week 25 of treatment using a procedure conducted in the previous research^13^.

### Tissue and serum sample

Brain tissue lysate for p16^INK4a^ protein levels and neurogenesis markers measurements were made in 1:10 (mg:µl solvent) in RIPA Lysis and Extraction Buffer (89900) (Thermo Scientific™) with 1% Halt™ Protease Inhibitor Cocktail (Thermo Scientific™) and 1% phenylmethylsulphonyl fluoride. Lysate was homogenized by short-burst cold sonication. The supernatant was obtained through 4 °C centrifugation at 15,000×*g* for 30 min. Serum for neuron-specific enolase (NSE) measurement was obtained after centrifuging clotted blood at 5000 rpm for 30 min.

Brain tissue lysates for inflammation and oxidative stress analysis were made in 1:10 (mg:µl solvent) using Phosphate Buffer Saline 0.05 M pH 7.2 as solvent. Homogenization and centrifugation procedures were as mentioned before. Total protein of tissue supernatant and serum were calculated using Walburg-Christian method. Bovine Serum Albumin 1% was used as the standard protein.

### Inflammation and oxidative stress analysis

#### (a) Inflammation analysis by measuring IL-6 protein

The inflammation marker IL-6 was analyzed through the competitive ELISA method based on the Rat Interleukin-6 ELISA Kit (FineTest^®^). A total of 100 µl of sample, standard and blank were put into the well in duplicate. The plate was then incubated for 90 min at 37 °C. Washing was carried out with a 1× wash buffer of 300 µl, repeated twice. A total of 100 µl of Biotin-labeled Antibody working solution was added to each reaction well. Incubation was then carried out for 60 min at 37 °C. Washing was done three times, with a 1 min immersion time for each round. A total of 100 µl of Streptavidin-HRP Antibody Conjugate (SABC) working solution was added to all reaction wells, and then incubation was carried out for 30 min at 37 °C. Washing was performed five times, with a 1 min immersion time for each round. A total of 90 µl of TMB substrate was added to all reaction wells, and then incubation was carried out for 15 min at 37 °C. A total of 50 µl of stop solution was then added to all wells. Absorbance was read at a wavelength of 450 nm.

### (b) Oxidative stress analysis

MDA was assessed as the parameter for lipid damage reflecting oxidative stress, while GSH and catalase-specific activity serve as indicators of endogenous antioxidant activity for coping oxidative stress. Will's method was used to determine the concentration of MDA. Tetrahydroxyprophane 1:80.000 in 50 nmol/ml concentration was standard in Will's method. Two hundred microliters of 20% trichloroacetic acid (TCA) were introduced into microtubes containing 400 µl of homogenate. Following centrifugation at 3000x*g* for 10 min, the resulting supernatants were transferred to clean 2 ml microtubes. Subsequently, 400 µl of 0.67% thiobarbituric acid (TBA) were added to each tube, and the samples were incubated in a 100 °C water bath for 10 min. After cooling to room temperature, the absorbance was measured at 530 nm. At this point, the reaction between MDA and TBA in acidic conditions resulted in a pink color. The MDA concentration was determined by correlating the absorbances of each sample with a standard linear curve represented by the equation y = ax + b, where y is the average absorbance and x is the MDA concentration in nmol/mg tissue^33^.

Ellman's method was used to determine the concentration of GSH in brain tissue supernatants. Reduced Glutathione in 2 mg/ml concentration was used as the standard in Ellman's method. Fifty microliters of the sample were mixed with 200 µl of 5% TCA in microtubes. After centrifugation at 3000×*g* for 10 min, the supernatants were carefully collected and transferred to new tubes. Subsequently, phosphate buffer at pH 8.0 was introduced to the tubes. Finally, 25 µl of DTNB were added to the sample tubes, and the samples were incubated in the dark for 1 h. Absorbances were then measured at 412 nm, and GSH concentrations were determined based on the linear curve derived from the glutathione standard^33^.

The determination of catalase-specific activity involved adding 1900 µl of H_2_O_2_ (at a ratio of 1:4000) to a 100 µl sample that had been previously placed in a UV cuvette. Blank samples were also prepared, consisting of 100 µl of 0.05 M pH 7 PBS and 1900 µl of H_2_O_2_ (1:4000). Absorbance readings were taken at the wavelength of 210 nm at the 30th and 150th seconds. The catalase activity was calculated using the following formula:

$$\mathrm{Catalase \,activity }({\text{U}}/{\text{ml}}) = \frac{(\Delta \mathrm{ Abs sample}-\Delta \mathrm{ Abs blank})/{\text{minute}}}{\mathrm{ H}_{2}{\text{O}}_{2}\mathrm{ molarity} \times {\rm sample \,volume}} \times \mathrm{ sample \,dilution}$$

The specific activity of catalase (U/mg protein) was determined by dividing the catalase activity by the protein level of the homogenate^33^.

### Cellular senescence analysis

Measurement of SA-β-Gal activity was carried out by fluorometric technique, according to the protocol of the 96-well Cellular Senescence Assay Kit [Cell Biolabs]. Treatment of samples, from the preparation of homogenates to the examination of SA-β-Gal activity, was carried out according to the kit protocol. According to manual instruction, a total of 25 µl of sample was reacted with 25 µl of 2 × assay buffer with the composition. Samples and reagents were reacted in a dark room at 37 °C for 3 hours. Fluorescence was read at an excitation wavelength of 360 nm and an emission wavelength of 465 nm. SA-β-Gal activity was calculated as the absorbance value divided by the total protein of the sample.

Measurement of p16^INK4a^ protein levels in samples was performed using the principle of competitive ELISA using the kit and precoated microplate Rat Antioncogene p16 protein ELISA Kit (MyBioSource). The preparation of sample homogenates through protein level measurements was carried out according to the kits' protocol. A total of 100 µl of sample, standard and blank, were put into the well in duplicate. A balance solution was added to the sample as much as 10 µl. A total of 50 µl of enzyme conjugate was added to the samples and standards. The plate was then incubated for 1 h at 37 °C. Washing was carried out with a 1× wash buffer of 300 µl, repeated 5 times. A total of 50 µl of substrates A and B was added to all reaction wells, and then incubation was carried out for 15 min in the dark at 37 °C. A total of 50 µl of stop solution was then added to all wells. Absorbance was read at a wavelength of 450 nm.

### Brain damage analysis

Neuron-specific Enolase/NSE was analyzed through the competitive ELISA method based on the protocol of the Rat NSE (Neuron-specific Enolase) ELISA Kit (FineTest^®^). Blood serum was used as the sample in this measurement. Serum preparation through analysis was performed according to the kit's manual. A total of 100 µl of sample, standard and blank, were put into the wells in duplicate. The plate was then incubated for 90 min at 37 °C. Washing was carried out with a 1× wash buffer of 300 µl, repeated twice. A total of 100 µl of Biotin-labeled Antibody working solution was added into each reaction well. Incubation was then carried out for 60 min at 37 °C. Washing was done 3 times, with a 1 min immersion time for each round. A total of 100 µl of Streptavidin-HRP Antibody Conjugate (SABC) working solution was added to all reaction wells, and then incubation was carried out for 30 min at 37 °C. Washing was performed 5 times, with a 1 min immersion time for each round. A total of 90 µl of TMB substrate was added to all reaction wells, and then incubation was carried out for 15 min at 37 °C. A total of 50 µl of stop solution was then added to all wells. Absorbance was read at a wavelength of 450 nm.

### Neurogenesis analysis

BDNF mRNA expression was analyzed using Quantitative Reverse Transcriptase Polymerase Chain Reaction (qRT-PCR)**.** Total RNA was isolated from the brain samples using the Quick RNA Miniprep Plus Kit (Zymo Research). Primers were designed using Primer3 (*GitHub*) and manufactured by *Integrated DNA Technology Singapore*. Primer sequences used were BDNF *Forward*: 5'-AAGGACGCGGACTTGTACAC-3' *Reverse* 5'-CGCTAATACTGTCACACACGC-3' and GAPDH *Forward* 5'-TCAAGAAGGTGGTGAAGCAG-3' *Reverse* 5'-AGGTGGAAGAATGGGAGTTG-3'^31^.

BDNF mRNA relative expression measurements were performed using the One-Step qRT-PCR working principle with the SensiFAST™ SYBR^®^ No-ROX One-Step Kit (BIO-72005) (Bioline). The master mix was prepared according to the kit's protocol. qRT-PCR was conducted in a 3-step cycling procedure at a 57 °C annealing temperature. Relative mRNA expression was calculated using Livak's equation. Measurement of protein levels of mature-BDNF, pro-BDNF, and ratio mature/pro-BDNF of brain samples was performed by the sandwich ELISA method using the Mature BDNF Rapid™ ELISA Kit: human, mouse and rat (Biosensis^®^) and the pro-BDNF Rapid™ ELISA Kit: human, rat and mouse (Biosensis^®^). A total of 100 µl of sample homogenate was reacted with antibodies at the bottom of the well for 45 min (m-BDNF analysis) and 90 min (pro-BDNF analysis) at room temperature (25 °C), on a shaker speed of 250 rpm. After washing, the sample was reacted with 100 µl of anti-matureBDNF antibody for 30 min (m-BDNF analysis) and anti-proBDNF for 45 min (pro-BDNF analysis), at room temperature (25 °C), on a shaker speed of 250 rpm. After washing, the sample was reacted with 100 µl of HRP-Streptavidin conjugate for 30 min at room temperature (25 °C), on a shaker speed of 250 rpm. After washing, the sample was reacted with 100 µl of TMB-substrate for 7 min at room temperature, in the dark, without shaking. 100 µl of stop solution was added, and the light absorption was read at a wavelength of 450 nm. The mature/proBDNF ratio is calculated based on the levels of m-BDNF and pro-BDNF produced in the ELISA process. Calculations are carried out using ratio and comparison formulas using the Excel program in Microsoft 365 (Microsoft^®^).

### Statistical analysis

Differences in each test group were measured by a parametric One-way ANOVA + post hoc Tukey test (when data were homogeneous) or Brown-Forsythe and Welch One-way ANOVA + post hoc Dunnett T3 (when data were not homogeneous) on normally distributed data. In data with a non-normal distribution, differences between groups were tested using Kruskal-Wallis non-parametric analysis + Bonferroni correction.

All data analyses were conducted using SPSS^®^ Version 29 (IBM^®^) software. The analyzed data were presented as mean ± standard error of the mean (SEM) or median (min-max) comparison graphs, created using GraphPad Prism 10 (Dotmatics) software. The significance of the data was indicated when the p-value < 0.05.

## Data Availability

The datasets employed and/or scrutinized in the present study can be obtained from the corresponding author upon reasonable request.
